# Roles of natural resources, globalization, and technological innovations in mitigation of environmental degradation in BRI economies

**DOI:** 10.1371/journal.pone.0265755

**Published:** 2022-06-24

**Authors:** Abdul Majeed, Chengang Ye, Ye Chenyun, Xu Wei, Muniba

**Affiliations:** 1 Business School, Huanggang Normal University, Huanggang, Hubei, China; 2 Business School, University of International Business and Economics, Beijing, China; 3 School of Accounting, University of Shandong Management, Jinan, China; 4 Accounting School, Hubei University of Economics, Wuhan, China; 5 School of Insurance and Economics, University of International Business and Economics, Beijing, China; Wroclaw University of Economics and Business: Uniwersytet Ekonomiczny we Wroclawiu, POLAND

## Abstract

The environmental issue has become a global problem that needs to be examined frequently, motivating researchers to investigate it. Thus, the present study has investigated the asymmetric impact of natural resources, technological innovation, and globalization on the ecological footprint in the presence of environmental Kuznets curve (EKC) in Belt and Road Initiative (BRI) economies. This research divided the BRI economies into high income, middle-income, and low-income levels to capture income differences. The study has used annual time series data from 1990 to 2018. The study applied a novel Augmented Mean Group estimators method to calculate the robust and reliable outcomes. The findings show that natural resources drastically damage the environment quality, whereas technological innovations are helpful in reducing environmental degradation. Moreover, the result of the interaction term (natural resources and technological innovations) negatively impacts the ecological footprint. Interestingly, these findings are similar in the three income groups. In addition, globalization improves environmental quality in the middle-income BRI economies but reduces in high-income, low-income, and full sample countries. Furthermore, the Environmental Kuznets Curve (EKC) concept has been validated across all BRI economies. In line with these findings, several relevant policies are recommended for a sustainable environment in the BRI economies.

## 1. Introduction

**Fig 1:**
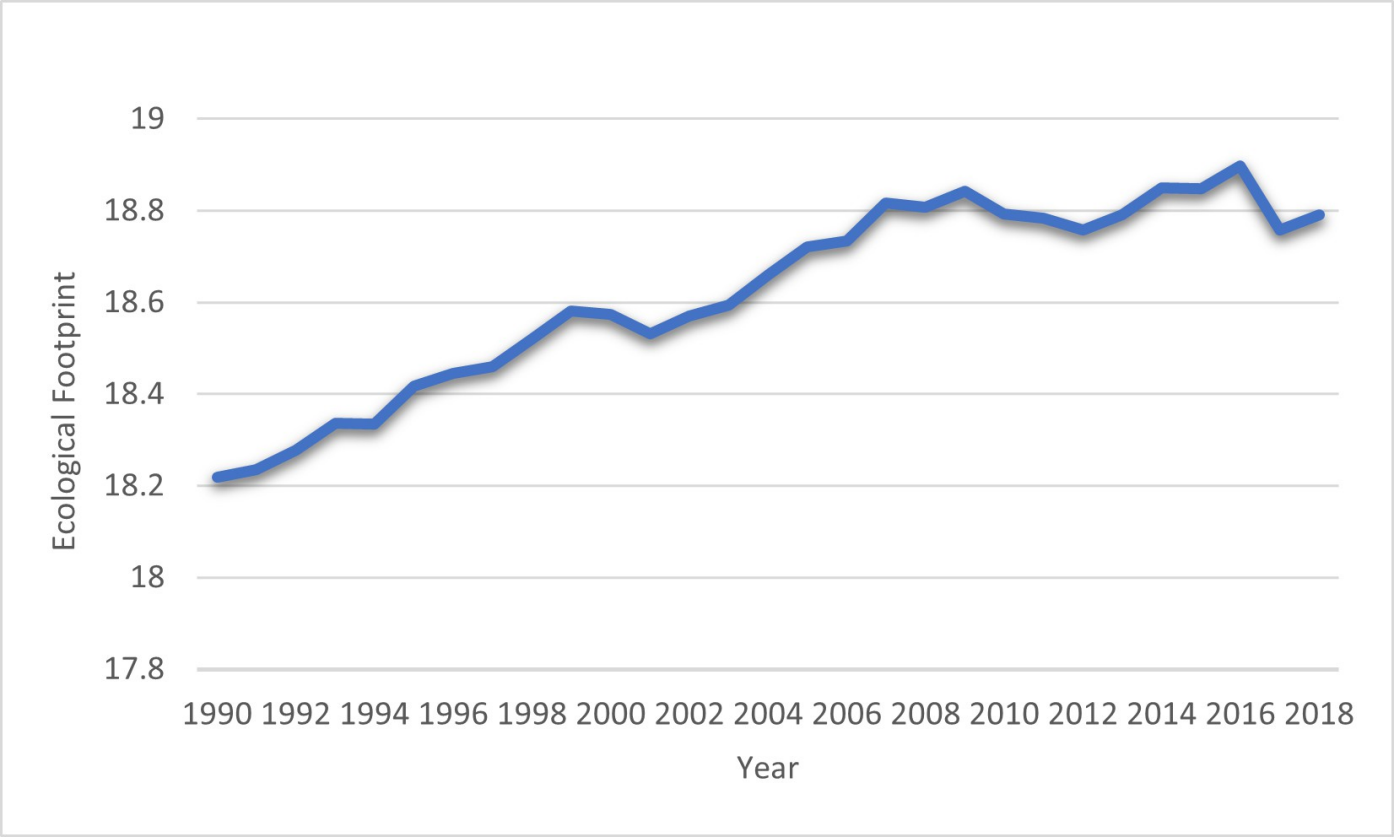
Ecological footprint in BRI economies: Source (Global Footprint Network).

The rising global warming trends have greatly interested policymakers in cleaning the environment using climate change mitigation strategies, and it seems to be a part of a broad consensus. In recent years, environmental conditions such as pollution, substandard sanitation, and significant loss of natural resources (NR) and forest reserves have been key concerns for the countries. Meager environmental conditions jeopardize human health and economic well-being. These elements are vulnerable to climate change, including health, natural and physical capital, and access to water, food, and land [1]. These environmental problems have sparked a worldwide campaign to resist climate change. However, in recent years, Belt and Road Initiative (BRI) economies have been straining efforts to upgrade their industrial movement, massive combustion of fossil fuel energy in the manufacturing sector, consequently increasing global warming [2]. Researchers have traditionally used carbon (CO_2_) emissions to proxy environmental quality in current environmental sustainability literature. However, this indicator is criticized by several scholars; CO_2_ emissions are accountable for a minor portion of the whole environment and do not fully encapsulate environmental pollution. Nathaniel and Khan [3] claimed that CO_2_ emission does not anticipate the stocks of resources (e.g., oil, soil, forest, gas, and petroleum). Therefore, it is necessary to use a proxy inclusive in modeling for environmental sustainability those imitators the limitation links with CO_2_ emission and offer suitable insight to policymakers/regular authorities related to the environment. For this situation, The ecological footprint (EF) is a widely recognized proxy for environmental quality that can manage and assess NR [4]. Hence recent empirical literature has used EF to measure environmental quality [3,5,6]. Fig 1 show the trend of EF in BRI economies from 1990 to 2018.

The NR has an essential element of the global economy, specifically in BRI economies that depend on extricating them for a significant portion of their economic growth [7]. The NR comprise forest, gas, oil, mineral, and coal. However, the link between NR and environmental degradation is intricate and owns contrasting evidence. As an example, Shen et al. [8]; Hussain et al. [9]; Udi et al. [10]; Wang et al. [11] have documented that NR rent positively influences environmental quality, Whereas Khan et al. [4]; Adedoyin et al. [12]; Li et al. [13]; Balsalobre-Lorente et al. [14] have described a negative association between NR and the environmental quality. Certainly, the literature on the association between NR and an inclusive environmental proxy like EF and additional inquiries are essential to moving towards a sustainable environment. NR is directly associated with the income level of an economy. In the first stage, people utilize more energy (e.g., more NR) for development purposes, which will increase the economic growth and ignore its effects on the environment, but in later stages, when the standard of living improves, than they adopt a cleaner environmental strategy, protection of NR and most concern on energy-efficient products indicating the presence of an Environmental Kuznets Curve (EKC). Hence, NR can significantly enhance environmental quality and boost economic growth [15].

Globalization (GLO) which is categorized by a decrease in barriers to the drive of technology, goods, physical and human capital, is a vital feature of indecisive (Gross Domestic Product) GDP and environmental sustainability [16]. The literature proposed that the GLO process can lead to economic growth Gurgul and Lach, [17], Atil et al. [18] as GLO links all the involved economies through foreign direct investment, trading, improve the efficiency of NR, technological transfer, and exchange of human and physical capital. However, researchers extensively studied the influence of GLO on environmental sustainability; they did not reach any consensus about the specific role of these variables in environmental quality. For example, Wang et al. [19], You & Lv [20] analyzed the impact of GLO on environmental performance and observed a positive effect of GLO on environmental quality. While Saud et al. [21], Akadiri et al. [22] observed the detrimental impact of GLO on environmental degradation remains an unresolved and evolving debate in the literature.

There are several compelling reasons to undertake this study in BRI economies. From the start of the BRI in 2013 through the end of 2019, China put 760 billion US dollars, with 39 percent going into the energy industry, roughly 26 percent going into transportation, and 7 percent going into metals [23]. In terms of NR, the BRI countries have 58.54 percent of proven reserves of crude oil, 53.82 percent of natural gas output, 74.69 percent of total coal output, and 55.17 percent of oil supplies worldwide [9]. Likewise, this project reaches 62% of the world’s population. These countries account for 31% of world Gross Domestic Product (GDP), and the share of global trade is 35% [24]. Furthermore, this project is accountable for 28% of CO_2_ emissions and a 20°^C^ increase in global temperature (excluding China). Therefore, assuming development proceeds as projected, CO_2_ emissions will increase by 66% until 2050 [25]. The BRI economies have critical economic significance because of their economic and global connectivity [26]. All these factors combine to make the BRI a viable option for research in environmental economics. To stimulate the economic growth and efficient utilization of NR, the efficient utilization of NR is necessary [27]. The green technological innovations can enhance the utilization and allocation of NR; it increases the capability of raw materials and also increases the exponential of NR to achieve the path of sustainable development [28]. Moreover, the GLO helps to enhance the efficiency of NR extractions using technological innovation (TE) [15]. So, the current study’s objective is to investigate whether NR, GLO, and TE foster environment quality?

This work contributes to the current literature in the following ways. First, this research examines the effect of NR, GLO, and TE on the EF from 1990 to 2018 for 90 BRI countries. Besides, this study divides the BRI countries into three income levels (i.e., high income, middle income, and low income) to examine the influence of these potential indicators on EF to assess potential disparity in the association between NR extraction and EF due to their income differences. Second, the present study used the moderating effect of NR with TE in reducing the EF. It would be helpful to examine whether NR indicated with TE reduces the overall level of EF in the BRI countries. This moderating effect may help to improve NR efficiency through TE [5]. Third, this research also examines the EKC hypothesis of BRI countries. Fourth, following confirmation of the possible cross-sectional dependence across cross-sections, this study used a comparatively advanced and robust econometric approach (i.e., CIPS unit root test, Westerlund cointegration approach, and augmented mean group for long-run elasticity), which enhances the efficiency and consistency of our finding. Finally, this study used the greenhouse gas (GHG) emission, another environmental proxy, and matched the outcomes to ensure robustness.

The rest of the paper is structured as seen below. Section two discussed the literature review of earlier studies. Section three explains the theoretical framework, data, and methodology. Section four discusses the findings and their interpretation and robustness checks. Finally, Section five reveals the conclusion and policy implications.

## 2. Literature review

Although several empirical studies investigate the NR- environment, TE-environment, and GLO-environment nexus separately, however, none of the studies examine these links simultaneously in a single model. Consequently, this study scrutinizes these relationships under separate titles and adds to the existing literature.

### 2.1. Nexus between natural resources (NR) and environment

Recently, environmental sustainability and NR have received more attention among policymakers and researchers. For example, Ahmad et al. [5] analyzed the relationship among NR, TE, GDP growth, and environmental degradation in twenty-two emerging countries from 1984 to 2016. The outcome suggested that NR and GDP growth increase environmental degradation, while TE has a favorable influence in reducing environmental deterioration. Similarly, Erdoğan et al. [29] inspected the dynamic association between NR, globalization, human capital, urbanization, and EF in twenty-three Sub-Saharan African countries covering 1980–2016. The results revealed that NR urbanization enhances environmental degradation, while globalization and human capital improve it. Likewise, Danish et al. [6] examined the relationship among renewable energy use, NR, urbanization, and environmental degradation in BRICS economies from 1992–2016. Their findings show that renewable energy, urbanization, and NR enhance environmental quality.

**Fig 2:**
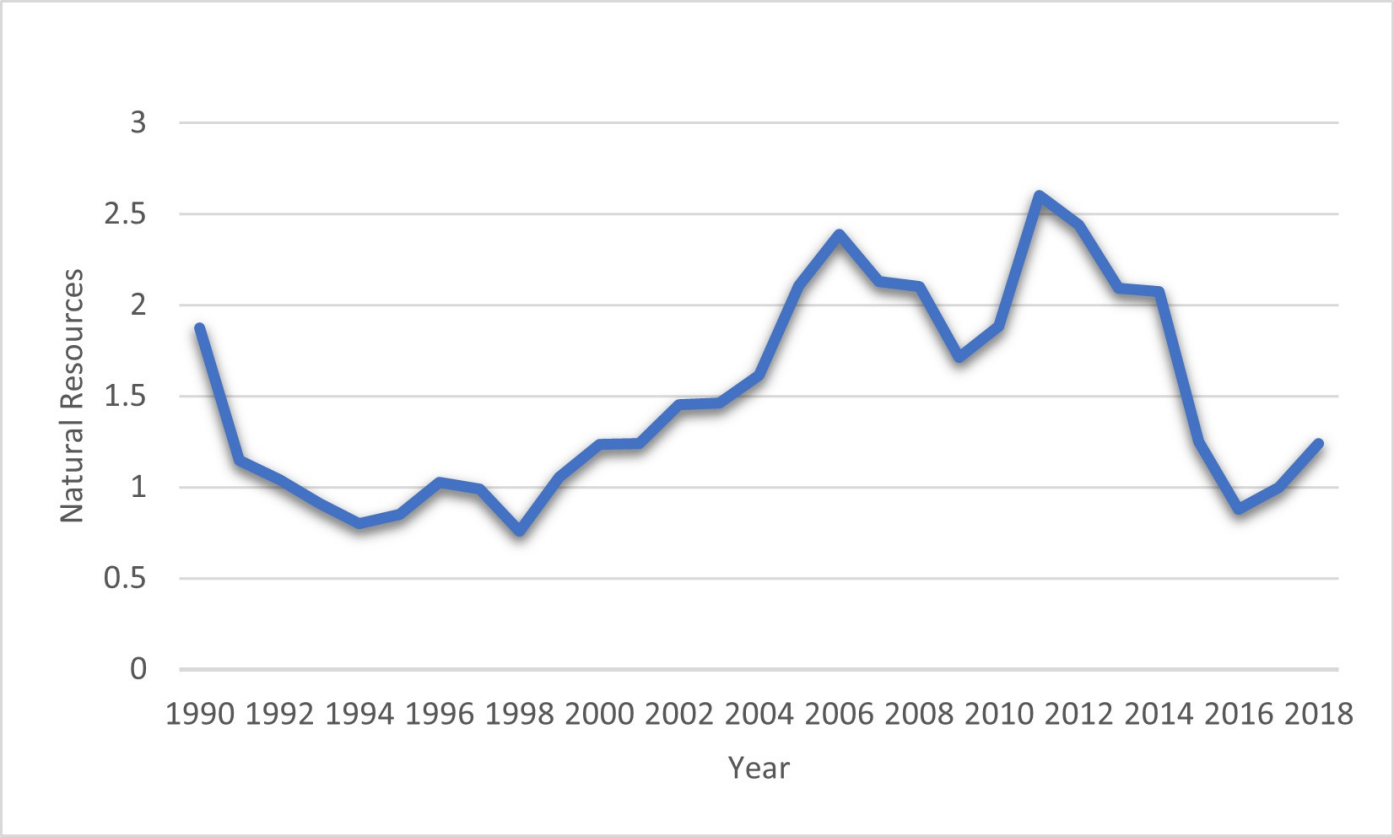
Natural resources in BRI economies: Source (World Development Indicators).

Balsalobre-Lorente et al. [14] analyzed the association among NR, GDP growth, renewable electricity based on five European Union countries from 1985–2016. The study results specified that renewable electricity and NR improve environmental sustainability. However, Khan et al. [30] investigated the NR, tourism, energy use, and environmental degradation nexus in 51 BRI economies from 1990–2016. The study’s findings revealed that NR is causally linked to tourism, energy use, and environmental degradation in these economies. Between the years 1970–2016, Ahmed et al. [31] revealed that NR and urbanization intensified the degradation of the environment. In contrast, Human capital has a positive effect on the environmental quality in the case of China. However, Zafar et al. [32] showed the negative relationship between NR and environmental deterioration due to eco-friendly technologies. To sum up, after discussing the literature review and focusing on the influence of NR on environmental sustainability, the effect of NR on the environment varies from country and time disparities. Fig 2 show the trend of NR in BRI economies from 1990 to 2018.

### 2.2. Nexus between technological innovation (TE) and environment

According to endogenous growth theory, research and development (R&D) expenditures can boost economic productivity and NR utilization, yet the involvement of TE in environmental sustainability, especially EF, is uncertain [33]. Chen and Lee [34] used the Ordinary Least Squares (OLS) and Fixed effect method to look into the connection between globalization, TE, and the environment in ninety-six economies from 1970–2016. The findings demonstrate that TE has a favorable effect in diminishing environmental damages. Kumail et al. [35] examined the dynamic relations between TE and environmental sustainability in Pakistan from 1990 to 2017. The findings explore that TE enhanced the environmental quality.

**Fig 3:**
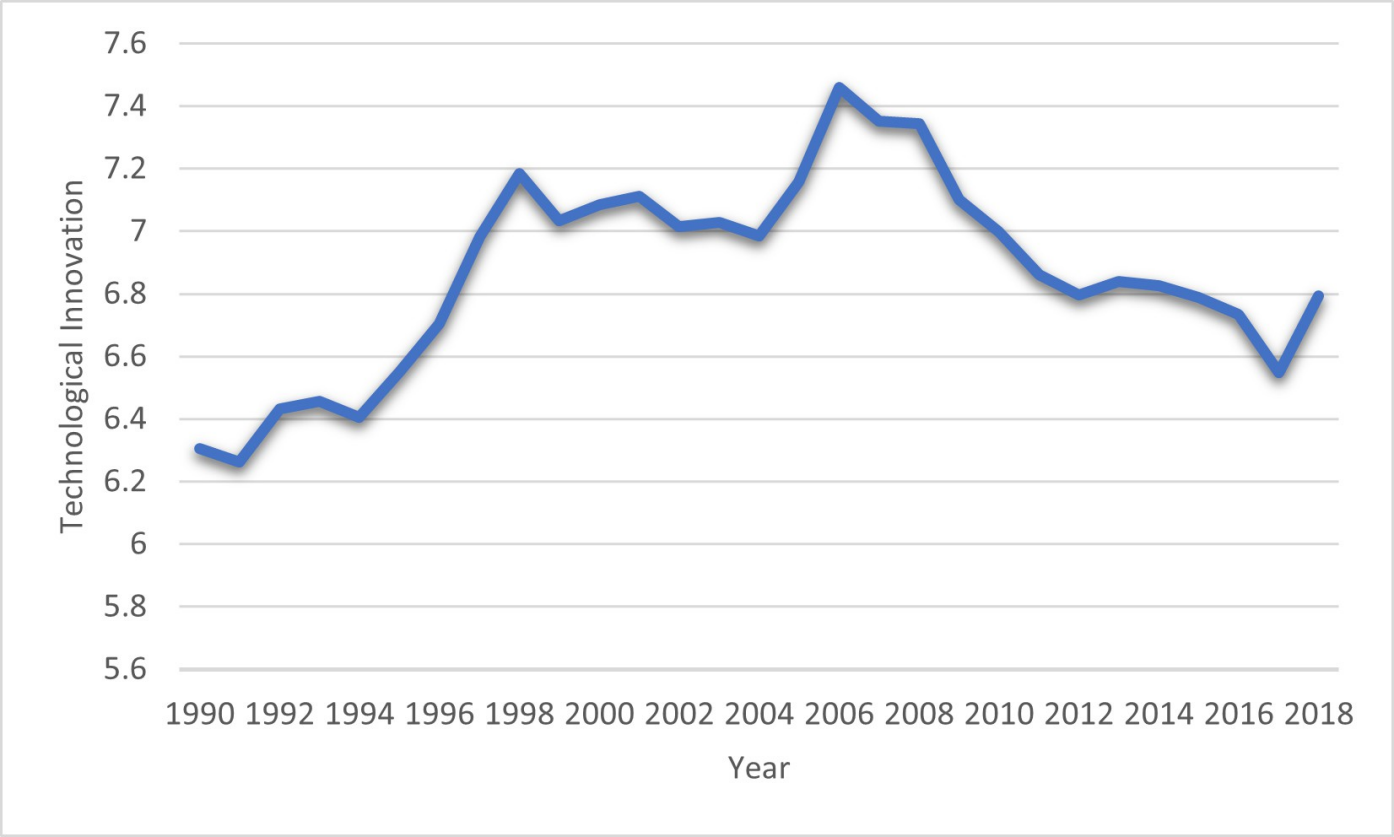
Technological Innovation in BRI economies: Source (World Intellectual Property Organization).

Likewise, Ke et al. [36] studied the causal link between the TE and EF for 280 Chinese cities from 2014 to 2018. The outcomes of this study revealed that TE increases the environmental quality. Ganda [37] examined TE and environmental degradation and found that TE enhanced the environmental quality through investment in the R&D sector. Most researchers believe that TE is favorable to minimizing environmental degradation [11,38–40]. They argued that TE introduces the efficient progression of new technological applications. Therefore, it directly enhanced energy efficiency and reduced fossil fuel energy utilization demand. Therefore, it improves the environmental quality. Alternatively, other researchers believed that TE might negatively impact environmental degradation [37,41–43]. Ikram et al. [44] reveal that green technology investments create different forms of value for the country’s economy. The value of investing in green technologies requires triggers related to tangible resources (e.g., financial capital). In summary, the impact of TE on environmental degradation is controversial that could be positive/negative, and academic literature still does not reach any definite conclusions. Fig 3 show the trend of TE in BRI economies from 1990 to 2018.

### 2.3. Nexus between globalization (GLO) and environment

Existing literature considered that GLO is the most vital indicator of environmental sustainability [21,45]. Theoretically, previous literature recognized the three channels of GLO that stimulate environmental sustainability, i.e., scale, composition, and technique effect [46]. Firstly, the scale effect is defined as when economic growth increases due to GLO will raise the volume of production that increases energy utilization and hence, increases environmental degradation in the region [47]. Secondly, the composition effect depends on the effect of GLO on environmental degradation due to variation in the economy’s industrial structure [48]. Finally, the technical effect denotes the numerous mechanisms by which GLO stimulates the amount of GHG by the industries and eventually reduces the environmental quality. These mechanisms contain eco-friendly technology that transfers from developed to developing countries due to their GLO process.

**Fig 4:**
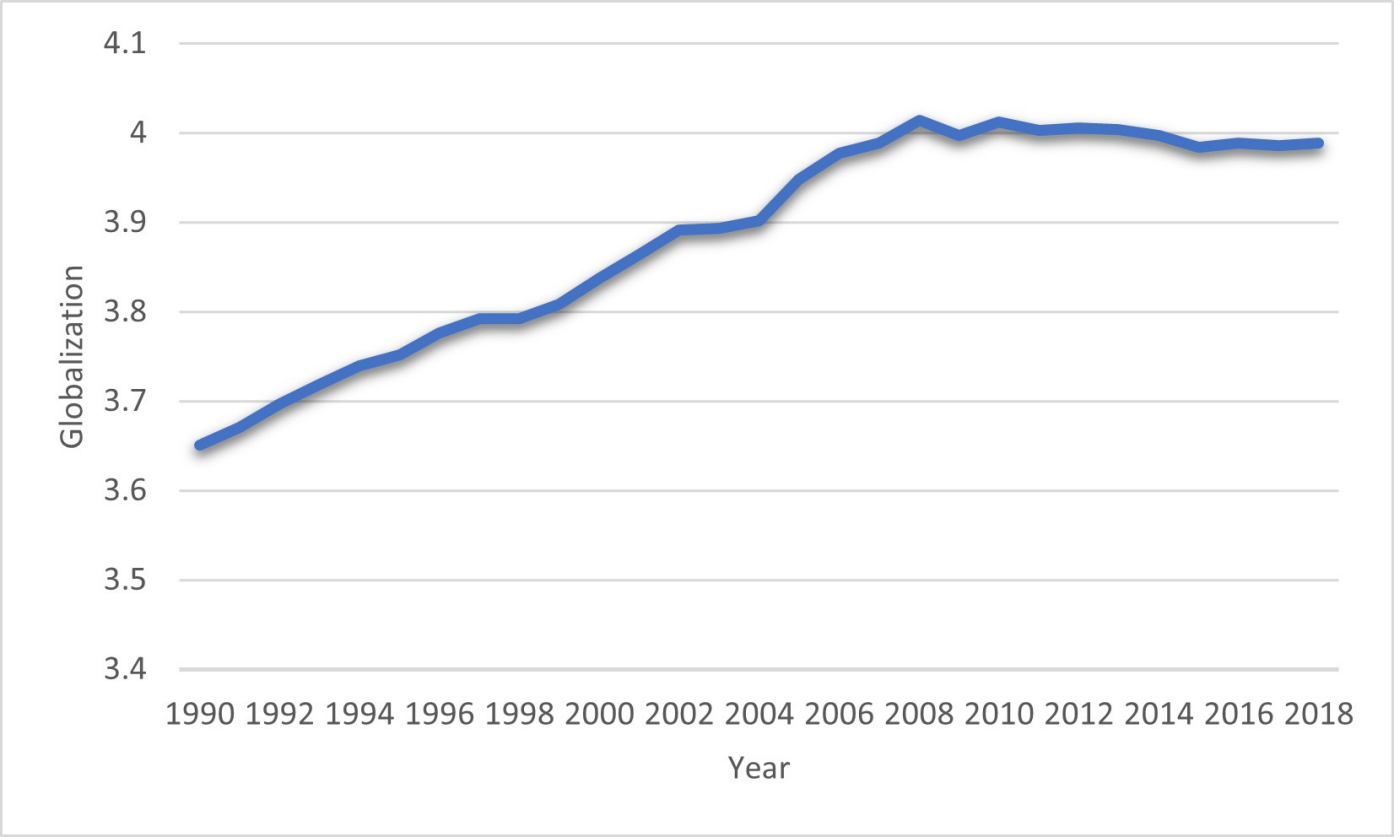
Globalization in BRI economies: Source (KOF globalization Index).

In this regard, several studies have found that GLO has a negative impact on environmental degradation [45,49–51]. The findings of these studies observed that the GLO process negatively influences environmental sustainability due to fewer environmental regulations because these developed economies shift their polluted industries resources in developing countries. Conversely, various researchers have found that GLO has an environmentally favorable effect [21,52]. They argued GLO brings eco-friendly technologies that enhance economic growth with fewer emissions and improve environmental quality. The literature review displays that GLO has a contrary influence on environmental sustainability, and theoretical literature does not reach any concurrence. Fig 4 show the trend of GLO in BRI economies from 1990 to 2018.

### 2.4. Nexus between economic growth and environment

**Fig 5:**
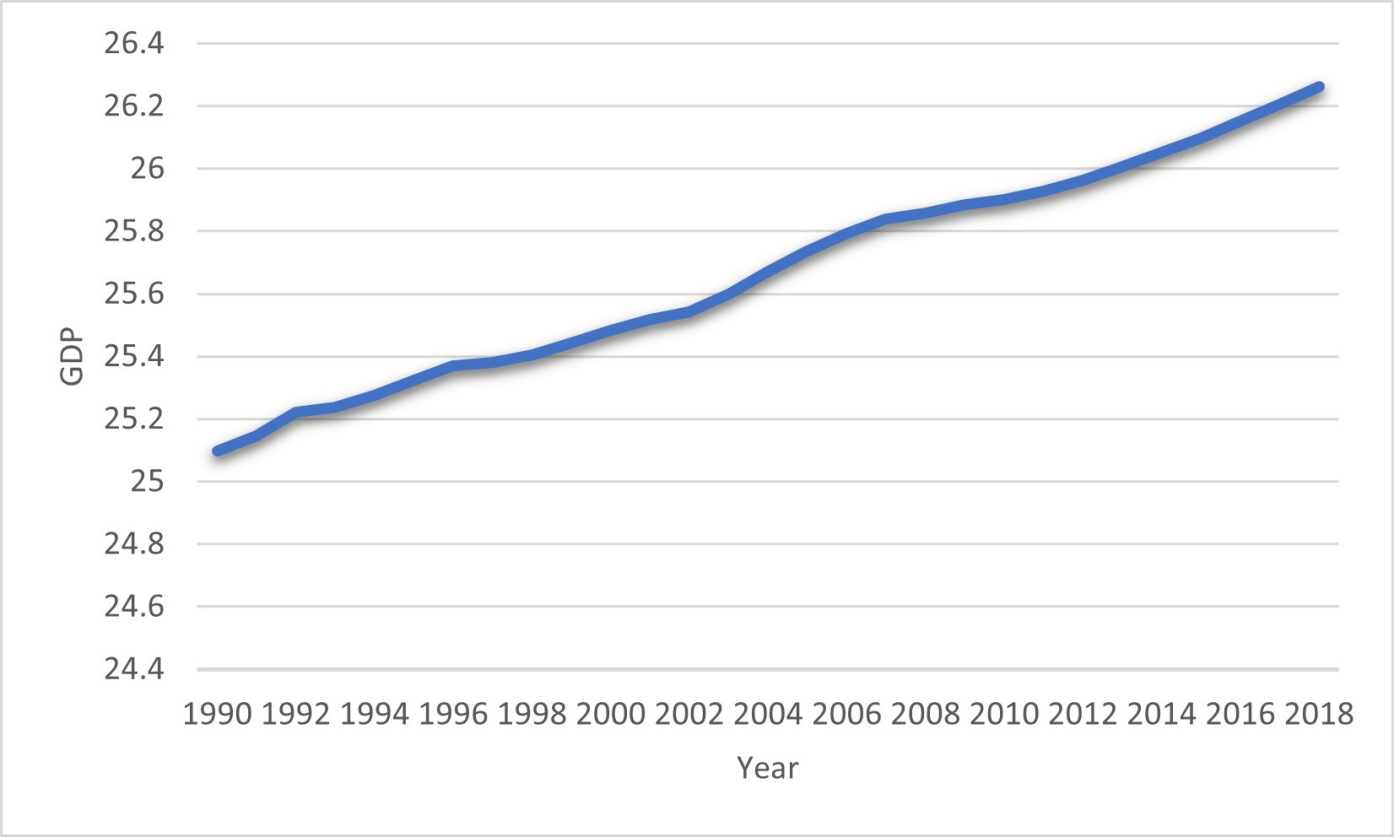
GDP in BRI economies: Source (World Development Indicators).

Since the pioneering work of Grossman and Krueger [53], the investigation regarding the association between economic growth and environmental deterioration centered on the EKC hypothesis supposing an inverted U-shaped influence of economic growth on environmental degradation. Further, Stern [54] suggested that if the level of real income enhances, it demands to improve environmental excellence because people will adopt the latest technologies in a production process to protect the environment. In this pursuit, several researchers found a U-shaped EKC hypothesis Danish et al. [6] for the BRICS economies, Ahmad et al. [5] for 22 emerging economies, and others have found inverted U-shaped EKC hypothesis, for instance, Wang et al. [55] for 150 economies, Altıntaş and Kassouri [56] for 14 EU economies, Destek and Sarkodie [57] for 11 economies, Bello et al. [58] for Malaysia and Khoshnevis Yazdi and Ghorchi Beygi [59] for 25 Africa countries, Ma et al. [60] for France and German country. Fig 5 show the trend of GDP in BRI economies from 1990 to 2018.

## 3. Theoretical framework, data, and estimation techniques

From a theoretical perspective, the association between NR, TE, GLO, and the EF on the treadmill production theory, endogenous growth theory, and ecological modernization theory. The treadmill theory of the product claims that environmental pollution directly affects NR and economic growth [61]. Endogenous growth and ecological modernization theories provide the idea that TE and GLO have more ability to support economies with sustainable economic growth in favor of environmental quality [5]. According to Spaargaren and Mol [62], ecological modernization theory was constructed on the principle of turning around how modern industrial societies deal with environmental problems. However, the rapid increase in production and economic growth reduces the NR and successively destroys environmental quality due to weak environmental regulations.

The EKC hypothesis is widely employed in the existing literature to examine environmental sustainability due to economic growth. It is assumed three different channels GDP growth affects environmental sustainability are scale effect, composition effect, and technique effect [63]. According to the scale effect, GDP has a negative influence on environmental quality where economic activities increase the volume of production level that will enhance environmental degradation. Further, the composition effect is based on the industry structure of an economy; for example, at the initial stages was environmental degradation with changes in economic reforms such as heavy industries (resource-intensive heavy manufacture industries). Finally, the technique effect recommends that outdated technologies are substituted by eco-friendly and updated capital that increases the environmental performance. Therefore, based on the EKC hypothesis, the negative influences of scale effects on the environmental quality will be dominant at the early stages of the economic growth process. However, the positive consequences of composition and technique effects lead to a decline in environmental degradation [54]. Using the assertions made above, we developed the following model to explain the influence of NR, TE, and GLO on the EF in the case of 90 BRI economies.


EF=f(NR,TE,GLO,GDP) (1)

The variables in the research are transformed into a natural logarithm. Following the pioneer study, Manning [64] recommended that normality problems be noticed in the variables before converting to logarithm form. The association between EF and other variables is inspected by employing the following model. Additionally, the study adds GDP square (GDP^2^) to test the EKC hypothesis’s validity. The EKC hypothesis in its extended form is presented as follows.


$$lnEF_{i,t}=\varphi_{0}+\varphi_{1}lnNR_{i,t}+\varphi_{2}lnTE_{i,t}+\varphi_{3}lnGLO_{i,t}+\varphi_{4}lnGDP_{i,t}+\varphi_{5}lnGDP^{2}{}_{i,t}+\varepsilon_{i,t}$$ (2)

In Eq 2, EF indicates the total ecological footprint, NR denotes natural resource abundance, TE presents the technological innovation, GLO demonstrates the globalization, GDP displays the economic growth per capita; *i* and *t* signify the 90 BRI economies and given time dimension (1990–2018) respectively. Therefore, this study aims that NR and Aside from its direct influence, TE may play a moderating role on the EF in 90 BRI economies. Thus, this study explores the moderation effects between NR and TE by focusing on this problem. Therefore, in Eq 3, we included a moderation effect term, which is written as follows:

$$lnEF_{i,t}=\varphi_{0}+\varphi_{1}lnNR_{i,t}+\varphi_{2}lnTE_{i,t}+\varphi_{3}lnGLO_{i,t}+\varphi_{4}lnGDP_{i,t}+\varphi_{5}lnGDP^{2}{}_{i,t}+\varphi_{6}lnNR*lnTE_{i,t}+\varepsilon_{i,t}$$ (3)

Where *φ*_0_ denotes the constant term, *ε*_*i*,*t*_ displays the error term and *φ*_1_ → *φ*_6_ represents the elasticity of candidate variables.

### 3.1. Data

This study explores the long-run association among NR, TE, GLO, and EF in 90 BRI economies using longitudinal data from 1990 to 2018. The name of the sample economies is provided in the Appendix section (see Table A). The classification of these countries is based on the UN countries’ classification [65]. The variable EF is calculated in terms of global hectares per capita, a total of carbon, farmland, built-up land, forest land footprint, fishing grounds, and grazing land. NR is the sum of forest rents, oil rents, coal rents, natural gas rentals, and mineral rents as a proportion of GDP. TE is determined by the number of patent applications. GLO is calculated as the political, social, and economic globalization index sum. The economic growth is computed in terms of constant 2010 US dollars. The GDP and NR data are acquired from the World Development Indicator [66]. The data on EF is extracted from Global Footprint Network [67], and GLO data is sourced from KOF globalization Index [68]. Lastly, the data of TE is collated from World Intellectual Property Organization [69].

### 3.2. Methodologies framework

This study has adopted the following advanced econometrics methods. (i) We confirmed the cross-sectional dependence (CD) by employing the Pesaran CD method. (ii) augmented cross-sectional IPS (CIPS) panel unit root methods are employed to verify the stationary level of candidate variables. (iii) The study used the Westerlund cointegration method to detect long-run relationships among variables. (iv) This research applied the AMG to examine the long-run elasticities of the variables.

**3.2.1.** Cross-sectional dependence (CD) test. The possible CD occurs due to externalities, geographical, globalization, spatial effects, economic integration, and individual-specific effects [5,48,70,71]. So, it is important to investigate the CD issue, whether it occurs or not, among all cross-sections. Moreover, in a panel data study, examining CD is critical because failing to do so may result in ambiguous and biased outcomes. Therefore, we employed a CD test to deal with the problem, such as the Pesaran CD test proposed by Pesaran [72].

$$CD=\sqrt{\frac{2T}{N(N-1)}}\sum_{i=1}^{N-1}\sum_{j=i+1}^{N}\rho_{ij}$$ (4)

where *T* signify time; *N* is the size of the panel data; and *ρ*_*ij*_ is the coefficient of correlation. The null hypothesis of the CD test is that there is no CD among the cross-sectional units. The alternative hypothesis is that CD exists among sample countries.

**3.2.2.** Slope homogeneity test. After evaluating the CD, the next step is to look at the slope homogeneity between the cross-sections. The issue of heterogeneity is critical due to differences in BRI countries’ demographic and economic structures. The consistency of panel estimators may be affected by variation in slope parameters. Because of this, this study employed the slope homogeneity approach proposed by Pesaran and Yamagata [73]. The test statistic’s equation is as follows:

$${\tilde{\Delta }}_{SH}=(N{)}^{\frac{1}{2}}(2K{)}^{-\frac{1}{2}}\left(\frac{1}{N}\tilde{S}-k\right)$$ (5)

$${\tilde{\Delta }}_{ASH}=(N{)}^{\frac{1}{2}}{\left(\frac{2k(T-k-1}{T+1}\right)}^{-\frac{1}{2}}\left(\frac{1}{N}\tilde{S}-k\right)$$ (6)

${\tilde{\Delta }}_{SH}$ represents the delta tilde and ${\tilde{\Delta }}_{ASH}$ shows the corrected delta tilde.

**3.2.4.** Panel stationarity test. The next phase in the econometric approach is to check the stationary/integration level of all involved variables after testing the CD of data. The first-generation unit root approaches like Levin-Lin and Chu, I’m, Pesaran, and Shin (IPS) can’t solve CD’s problem [48]. As a result, to account for the existence of CD, this research employed the second generation CIPS Pesaran [74]. The following are the CIPS test statistics:

$$\Delta CA_{i,t}=\varphi_{i}+\varphi_{i}Z_{i,t-1}+\varphi_{i}{\bar{CA}}_{t-1}+\sum_{l=0}^{p}\varphi_{il}\Delta \bar{CA_{t-1}}+\sum_{l=0}^{p}\varphi_{il}\Delta CA_{i,t-1}+\mu_{it}$$ (7)

Where ${\bar{CA}}_{t-1}$ and $\Delta \bar{CA_{t-1}}$ are the averages of the cross-sections. The statistics of the CIPS test are detailed in the study, as seen below:

$$\hat{CIPS}=\frac{1}{N}\sum_{i=1}^{n}CDF_{i}$$ (8)

Panel cointegration test. The next phase in the econometric process is to assess the long-run association between the variables. Westerlund [75] developed the second-generation cointegration test to find a long-run connection between the series. This test is superior to traditional cointegration approaches such as Kao and Pedroni as it gives unbiased estimates in the existence of CD and heterogeneity [76]. Westerlund tests comprise four types of test statics such as Gt, Ga (group), and Pt, Pa (panel), which is estimated through Eq 9 described as follows:

$$\alpha_{i}(L)\Delta y_{it}=\delta_{1i}+\delta_{2i}t+\alpha_{i}(y_{it-1}-\beta_{i}^{\prime }x_{it-1}+\lambda_{i}(L{)}^{\prime }v_{it}+e_{it}$$ (9)

Where $\delta_{1i}=\alpha_{i}(1)\phi_{2i}-\alpha_{i}\phi_{1i}+\alpha_{i}\phi_{2i}$ and *δ*_2*i*_ = −*α*_*i*_*ϕ*_2*i*_.

In Eq (9) *β*_*i*_ is a coefficient of error correction and *α*_*i*_ is the direction of the cointegration relationship between x and y. The following are the test statistics:

$$G_{t}=\frac{1}{N}\sum_{i=1}^{N}\frac{\alpha_{i}^{\prime }}{SE(\alpha_{i}^{\prime })}$$ (9.1)

$$G_{a}=\frac{1}{N}\sum_{i=1}^{N}\frac{T\alpha_{i}^{\prime }}{\alpha_{i}^{\prime }(1)}$$ (9.2)

$$P_{t}=\frac{\alpha^{\prime }}{SE(\alpha^{\prime })}$$ (9.3)

$$\alpha^{\prime }=\frac{P_{a}}{T}$$ (9.4)

In Eq (9), the parameter for error correction (*α*′) is calculated by putting the value of *P*_*a*_ = *Tα*′ as a result, the error correction variable can be defined as $(\alpha^{\prime })=\frac{P_{a}}{T}$ shows that If there is a short-run disequilibrium, the proportion of error should be adjusted annually.

**3.2.5.** Long-run estimation. Following that, we examine the long association between the NR and EF in the existence of TE, GLO, and economic growth. Economists suggest a number of methodologies for analyzing panel data. However, previous research used first-generation cointegration approaches (FMOLS, DOLS, ARDL, and so on), which might lead to biased outcomes in the existence of CD and heterogeneity. Thus, the AMG technique was used in this study Eberhardt [77]. This method is worthy for a variety of reasons. This technique is appropriate in the case of endogeneity, non-stationary, CD, and heterogeneity. Furthermore, this method takes into account correlation, particularly among cross-sections. The AMG equation is as follows:

$$\Delta EF_{it}=\varphi_{0}+\varphi_{1}\Delta NR_{it}+\varphi_{2}\Delta {TE}_{it}+\varphi_{3}\Delta {GLO}_{it}+\varphi_{4}\Delta GDP_{it}+\sum_{t=2}^{T}p_{t}(AD_{t})+\mu_{it}$$ (10)

Where, *AD*_*t*_ indicates the first difference *T*−1 dummies for the time, *j* specifies dummy time parameters. The next step is to *p*_*t*_ is substituted with *τ* variable, demonstrating the standard dynamic process as:

$$\Delta EF_{it}=\varphi_{0}+{\varphi_{1}\Delta NR_{it}+\varphi_{2}\Delta {TE}_{it}+\varphi_{3}\Delta {GLO}_{it}+\varphi }_{4}\Delta GDP_{it}+d_{1}(\lambda_{t})+\mu_{it}$$ (11)

$$\Delta EF_{it}-\lambda_{t}=\varphi_{0}+{\varphi_{1}\Delta NR_{it}+\varphi_{2}\Delta {TE}_{it}+\varphi_{3}\Delta {GLO}_{it}+\varphi }_{4}\Delta GDP_{it}+\mu_{it}$$ (12)

First, the group-specific regression model was reformed with *φ*_*t*_ and following that, the averages of group-specific models are calculated. This research applied the Common Correlated Effect Mean Group (CCEMG) approach to test the robustness of the model Pesaran [78].

## 4. Results and discussion

The first econometric step of empirical analysis scrutinizes the existence of CD among the variables. The results of CD tests of the null hypothesis (H_0_) of no CD between the variables are given in Table 1. According to the CD test proposed by Pesaran [72] is rejected at the 1% significance level. This specifies that an erratic shock (positive/negative) in one country will affect the other countries in the BRI region. The CD was further corroborated by absolute mean values ranging from 0.447 to 0.819. In contrast, BRI economies show a heterogenous slope due to the different growth patterns. As shown in Table 2, the BRI economies’ panel has various degrees of technological advancement and development. As a result, the slope homogeneity test findings indicate that the model has a data heterogeneity problem.

**Table 1:** Cross-sectional dependence test results.

Variables	Statistic	P-value	abs(corr)
EF	11.238***	0.000	0.480
NR	25.273***	0.000	0.687
TE	27.873***	0.000	0.447
GLO	20.289***	0.000	0.457
GDP	64.525***	0.000	0.815
GDP^2^	64.820***	0.000	0.819

Note: P<0.01, 0.05, 0.10 indicate ***, ** and *, respectively.

**Table 2:** Results of slope homogeneity test.

Test	BRI (full panel)		High-income		Middle-income		Low-income	
	Value	P-value	Value	P-value	Value	P-value	Value	P-value
$\tilde{\Delta }$	17.899***	0.000	15.899***	0.000	15.523***	0.000	14.273***	0.000
${\tilde{\Delta }}_{\mathrm{adjusted}}$	18.601***	0.000	16.548***	0.000	16.157***	0.000	15.217***	0.000

Note: P<0.01, 0.05, 0.10 indicate ***, ** and *, respectively.

After testing the CD, there is a dire requirement to find the integration order /stationary level of the variables. To do this, we applied CIPS unit root tests. Table 3 shows the CIPS unit root test outcomes revealed that EF, NR, and GLO are not stationary at levels representing that they cannot reject the null hypothesis. However, they become stationary at their first difference at a 1% significance level. These findings reveal that all the candidate variables are stationary, and it is appropriate to assess the long run cointegration of variables.

**Table 3:** CIPS panel unit root test result.

Variable	I(0)		I(1)		Order
	Intercept	Intercept & trend	Intercept	Intercept & trend	
EF	-1.724	-1.832	-3.511***	-3.735***	I(1)
NR	-1.689	-1.818	-3.473***	-3.706***	I(1)
TE	-2.909***	-3.145***	**−**	**−**	I(0)
GLO	-1.497	-1.499	-4.354***	-4.737***	I(1)
GDP	-2.355**	-2.742**	**−**	**−**	I(0)
GDP^2^	-2.103	-2.769**	**−**	**−**	I(0)

Note: P<0.01, 0.05, 0.10 indicate ***, ** and *, respectively.

In order to verify the long-run equilibrium between the variables, we used the second-generation test, namely Westerlund [75]. Table 4 explores the Westerlund cointegration test outcomes. The findings of the Westerlund tests show that all four models have a long run cointegration association. The outcomes in the high- and middle-income BRI region demonstrate robust likelihood values; they fail to reject the null hypothesis (H_0_) of no cointegration for Ga. In contrast, the outcomes for G_t_, P_t,_ and P_a_ provide an appropriate indication to reject the H_0_ with corresponding probability levels that are significant. Therefore, it shows that all variables comprise a long run cointegration.

**Table 4:** Westerlund panel cointegration test results.

	*G*_*t*_	*G*_*a*_	*P*_*t*_	*P*_*a*_
BRI (full panel)	-4.165*** [-9.507]	-12.925** [-1.916]	-17.616*** [-8.198]	-14.899*** [-5.374]
High-income	-4.238*** [-9.824]	-11.754 [-1.179]	-16.021*** [-6.939]	-13.850*** [-4.732]
Middle-income	-3.565*** [-9.824]	-11.057 [-0.741]	-14.191*** [-5.494]	-13.426*** [-4.472]
Low-income	-4.453*** [-10.757]	-13.212** [-2.096]	-20.755*** [-10.675]	-14.376*** [-5.053]

Note: P<0.01, 0.05, 0.10 indicate ***, ** and *, respectively. [] is for Z-value.

We can assess the long run relationship via the AMG method after completing the cointegration examination between the variables. The results of the AMG test are demonstrated in Table 5. The NR and the EF have a positive and significant association in Model 1 for the BRI full panel. Accordingly, a 1% increase in NR in BRI economies leads to driving an EF of about 0.449%. The average share of NR in BRI economies has increased proximately 1.968% from the years between 1990 to 2018 based on the rents for oil, natural gas, coal (hard and soft), mineral rentals, and forest rents [79]. These BRI economies are putting stress on NR reserves to achieve energy demand, enhancing the pressure on the environment. Considering these facts, we conclude that investment in clean energy (eco-friendly technology) should be an essential element in reducing the EF. The elasticity of TE is also significant and negative influence on EF, demonstrating that a 1% change in TE is associated with a 0.087 percent decrease in EF. Thus, the TE is a significant element for sustainable development, attaining energy efficiency, and supporting a low EF. These results align with [5,80].

**Table 5:** Results of AMG.

Variables	BRI (full panel) (1)	BRI (full panel) (2)	High-Income (1)	High-Income (2)	Middle-Income (1)	Middle-Income (2)	Low-Income (1)	Low-Income (2)
NR	0.449*** [0.134]	0.488*** [0.126]	0.420*** [0.142]	0.480*** [0.060]	0.212*** [0.068]	0.2752 [0.2758]	0.199*** [0.071]	0.314*** [0.044]
TE	-0.087** [0.036]	-0.084** [0.035]	-0.067* [0.039]	−0.068** [0.032]	-0.050** [0.021]	-0.042** [0.018]	-0.044* [0.024]	-0.021*** [0.007]
GLO	0.042*** [0.019]	0.070** [0.027]	0.047** [0.019]	0.044** [0.020]	-0.026** [0.011]	-0.043** [0.016]	0.034** [0.014]	0.011*** [0.003]
NR*TE	---	-0.039* [0.024]	---	-0.023*** [0.007]	---	-0.027* [0.015]	---	-0.014*** [0.005]
GDP	0.876*** [0.134]	0.852*** [0.148]	0.827*** [0.125]	0.786 ** [0.365]	0.497*** [0.082]	0.449*** [0.077]	0.519*** [0.099]	0.480*** [0.060]
GDP^2^	-0.029*** [0.006]	---	-0.023*** [0.008]	---	-0.018*** [0.005]	---	-0.018*** [0.007]	---
Constant	-2.184** [0.848]	-3.029*** [1.007]	-2.558*** [0.978]	-2.525** [1.172]	-2.353** [1.108]	-2.831*** [1.057]	-2.447* [1.359]	-2.227** [1.133]

Note: P<0.01, 0.05, 0.10 indicate ***, ** and *, respectively. [] is for standard error.

The coefficient of GLO has a positive impact on the EF, so the 0.042% change in the EF is due to GLO. Therefore, the policymakers should encourage foreign direct investment (FDI) to encourage investors to bring eco-friendly technologies and pollution-free industries. These findings coincide with [49]. Furthermore, in Model 1 of BRI countries, GDP and GDP^2^ positive and negative values with EF indicate the EKC hypothesis’s validity with an inverted U shape. Specifically, a 1% upsurge in GDP enhances the EF by 0.876%, while a 1% rise in GDP^2^ lessens the EF by 0.029%. So, maintaining a competitive advantage requires initiating positive changes in a diverse world regarding economic development and environmental degradation [81,82]. Ikram et al. [83] research findings show that governments must take corrective measures to prevent the economies from more damages and improve their logistics, environmental and quality performance. These findings align with [8,21]. According to Model 2 of the BRI full panel, the negative coefficient of the interaction term (NR* TE) shows that TE negatively moderates the association between NR in reducing EF, which means when NR improves the environmental quality due to promotion of TE.

Following Model 1 of high income BRI economies, the coefficient of NR has a significant positive impact on EF at a 1% significance level, indicating that a 1% increase in NR results in a 0.420 percent rise in EF. Since 2000, overall energy demand has grown by more than 83%, and the share of this development has been happened by a doubling consumption of fossil fuel utilization in BRI economies. Oil is also the primary source of power generation/energy demand in this region, leading to enhanced degradation of the environment. This subset of results is discovered similarly [4,6,12]. Alternatively, In the case of high income, the coefficient value of TE has a negative and statistically significant impact in BRI economies; particularly, A 1% rise in TE results in a 0.067% drop in EF. Specifically, TE comprises the development of new ideas, adjustment/modification of the current production process, and an essential solution for sustainable development and environmental issues [84]. Our results are consistent with the finding of [85].

Additionally, a 1% rise in GLO will result in a 0.047 percent increase in the degradation of the environment in the long run. This empirical evidence indicates that GLO enhanced the EF because most BRI countries rely on fossil fuels. These findings are in accordance with [21,22,46]. Conversely, GDP and GDP^2^ elasticity are (0.827 and -0.023) respectively; this supports the EKC hypothesis with a U shape. It is found that a 1% rise in GDP corresponds to a 0.827 percent surge in EF, while a 1% rise in GDP^2^ decreases EF by -0.023%. It means pollution level increases during the early stages of economic growth. However, after arriving at a specific point, the pollution level will decrease. This result is congruent with the findings of [5,86]. Moreover, according to Model 2 of the high income BRI economies, the negative coefficient of an interactive term (NR* TE) shows that TE negatively moderates the relationship between NR and EF.

It is noted that a 1% upsurge in NR* TE led to a 0.023% reduction in EF. In this regard, the result of NR in middle income BRI economies has a significant and positive effect on the EF in Model 1. Specifically, at the 1% significance level, a 1% rise in NR enhances the EF by 0.212 percent in the long run. This finding supports the hypothesis that NR is the primary source of environmental damage. The outcome supports the hypothesis that NR is the primary source of environmental damage in the region. The positive impact of NR can be supported by the fact that middle-income countries have the greatest number of oil-producing economies. These countries are placing immense pressure on their NR to meet their energy needs, causing environmental degradation. The findings of our empirical evidence are consistent with those of [5].

Furthermore, The TE coefficient has a negative and significant effect on EF, revealing that a 1% rise in TE reduces the EF by -0.050%. Thus, the TE outcomes minimized the EF through eco-friendly technologies and supported the existing findings Wang et al. [11,87] in contrast with [5]. The elasticity of GLO is also significant and negative, indicating that a 1% influence in GLO reduces the EF by 0.026%. This outcome is similar to those found by [21,52]. In order to support this result, Ahmad et al. [86] suggested that GLO brings environmentally friendly technologies and current inventive production practices, which boost economic growth while lowering EF.

Likewise, the EKC hypothesis’s validity is expressed by the positive and negative values of GDP and GDP^2^ on EF in middle income BRI countries. The findings are consistent with those of [5,88]. Moreover, Model 2 of middle income BRI countries show the significant moderation effect of NR with TE on EF. The outcome of the interaction term (NR* TE) reveals that it has a significant and adverse effect on the EF. Notably, a 1% rise in NR* TE results in a -0.027 percent decrease in EF. Ahmad et al. [5] contended that economic activities and trade openness enhance energy utilization, and revolutions in technology improve the efficient utilization of NR and energy efficiency; thus, it helps mitigate EF.

Considering Model 1 of low income BRI economies, the NR coefficient affects EF positively and significantly. It is worth noting that a 1 percent increase in NR outcomes 0.199 percent rise in EF. The low income BRI economies hold almost 20% of the world’s proven oil reserves and put massive pressure on their NR assets to accomplish their energy demand, enhancing environmental degradation [6]. These results are also in line with [5]. The concept of the inverted U-shaped EKC hypothesis is expressed by the positive and negative values of the GDP and GDP^2^ coefficients with EF. It is worth noting that a 1% increase in GDP results in a 0.519% rise in EF, whereas a 1% upsurge in GDP^2^ results in a 0.018% decline in EF. These results are comparable with [89]. Accordingly, in Model 2 for low income BRI countries, the result of the interactive term (NR* TE) has a significantly negative impact on EF, demonstrating TE could improve the utilization of NR and enhance the environmental quality through eco-friendly technologies. More specifically, a 1% influence in NR* TE will decrease the EF by 0.014%. However, we observed that NR (without interaction with TE) is significant in all models except Model 2 of middle income BRI countries, with no significant association with EF in contrast (with interaction term); it appears to have a significant influence on EF.

### 4.1. Robustness check

The robustness of the results mentioned above tested using the alternative measure of EF with GHG as a dependent variable and an alternative estimator, i.e., CCEMG [78]. According to Table 6, the results of NR are significantly lowering the quality of the environment while TE enhances environmental sustainability. In addition, in the case of a full panel of BRI countries, the findings likewise corroborate the inverted U-shaped EKC hypothesis. Furthermore, the interaction term between NR and TE (NR* TE) is a statistically negative influence on GHG emissions, similar to the AMG estimators’ findings. Hence our results are robust and reliable to both robustness checks (alternative variable and method), which ensure the accuracy of our findings.

**Table 6:** Robustness checks (Full Sample).

	Model 1			Model 2		
Variables	GHG	Std. Err.	P-value	GHG	Std. Err.	P-value
NR	0.632**	0.271	0.019	0.591**	0.240	0.014
TE	-0.065***	0.010	0.000	-0.032***	0.009	0.000
GLO	0.045***	0.016	0.010	0.041**	0.017	0.025
NR*TE	--------			-0.081***	0.020	0.001
GDP	0.743*	0.390	0.061	0.762**	0.310	0.014
GDP^2^	-0.091***	0.031	0.009	---------		
Constant	-2.598***	0.699	0.000	-2.696***	0.588	0.000

Note: P<0.01, 0.05, 0.10 indicate ***, ** and *, respectively.

## 5. Conclusion and policy implications

### 5.1. Conclusion

Emission forecasting is vital for global policymaking and emission reduction goals [90]. The cleaner environment notion is still emerging, valuable in current policies and agendas. For the past couple of decades, a sustainable environment has been a desired state worldwide. Environmental pollution can occur as a result of a variety of economic activities. Various socio-economic issues have a positive or negative impact on the environment. Many earlier studies have resulted in efficient input allocation as an environmentally beneficial component. This research explores the impact of NR, TE, and GLO with the interaction term (NR* TE) on the EF from 1990–2018 in BRI countries in the EKC hypothesis framework. Further, to capture the effect of income differences, this study divided the BRI countries’ samples into three income groups. The EF, a complete proxy (based on six different environmental indicators), quantifies environmental degradation in the current study [5,6,25,86]. Pesaran CD technique used to check the CD. The stationary level is investigated using the CIPS panel unit root technique.

The cointegration method, developed by Westerlund [75], determines whether there is a long-run link among the variables under consideration. The AMG estimator is employed in this research to estimate the long-run elasticity of variables. The findings present that an NR has a positive effect on EF while TE and interaction with NR (NR* TE) negatively impact the EF in the BRI countries. Interestingly, the same outcomes were found in three income groups regarding the association between NR, TE, and interaction terms but at a different magnitude and significance level. This finding reveals that NR reserves to meet energy demand, putting further strain on the environment. Given these facts, we infer that investing in clean energy (environmental technologies) should be a key component in lowering the EF. Furthermore, results confirm the income-wise EKC hypothesis for BRI countries.

According to the findings, this research recommends the following policy implications to stakeholders, governments, and policymakers in general, and specifically to BRI economies for environmental sustainability.

■ Policymakers in BRI countries should move resources away from resource-rich sectors of industries/manufacturing sectors to enhance/promote economic growth and use these NR efficiently for a progressive, sustainable environment.
■ TE should be utilized massively to use energy and NR efficiently.
■ The GLO process should not be ignored in the policy framework for a sustainable environment in BRI economies. A balance should be struck between the economic benefits of GLO and environmental deterioration. Furthermore, the policymakers should encourage clean and green foreign investment and welcome those investments, which carry technical skills, environmentally friendly technologies, and carbon-free methods in the BRI economies.
■ These countries should change their dirty energy strategy into renewable energy sources. The government of these countries should focus on trade promotion with the advanced technology by supplying eco-friendly technology and sources of renewable energy, including hydropower, solar, wind, biomass, geothermal heat, waste-based energy, and employ environmental regulation policies such as imposing carbon tax/quota system on emission-intensive products which could help to minimize environmental degradation. Environmentally friendly technologies will preserve the BRI countries’ international capacities while ensuring long-term environmental sustainability.
■ The enormous increase in EF and the economic performance of various economies continue to pique the interest of academics and practitioners. Concerns about global warming and its impact on human and animal health, hence sustainable development, are also growing. As a result, policymakers and academics must examine the critical significance of absorptive capacity in promoting sustainable development.

This research has limitations that should be addressed in future research. Because our analytical approach does not take into account crucial cultural and social aspects, future researchers could expand on this research by investigating the interaction role of institutional quality and NR in the pollution haven or halo hypothesis framework, making a significant contribution to the literature. It will ultimately help to control environmental degradation.

## Appendix: Table A

**Table:** 

List of BRI countries						
Sr #	Name of Country	Income Level		Sr #	Name of Country	Income Level
1	Albania	MI		46	Luxembourg	HI
2	Algeria	MI		47	North Macedonia	MI
3	Angola	LI		48	Malaysia	MI
4	Armenia	MI		49	Maldives	MI
5	Austria	HI		50	Malta	HI
6	Azerbaijan	MI		51	Moldova	LI
7	Bahrain	HI		52	Mongolia	LI
8	Bangladesh	LI		53	Morocco	LI
9	Belarus	MI		54	Mozambique	LI
10	Benin	LI		55	Namibia	MI
11	Bolivia	LI		56	Niger	LI
12	Bulgaria	MI		57	Nigeria	LI
13	Cambodia	LI		58	Oman	HI
14	Cameroon	LI		59	Pakistan	LI
15	Chile	HI		60	Panama	HI
16	Congo, Dem. Rep.	LI		61	Peru	MI
17	Costa Rica	MI		62	Philippines	LI
18	Croatia	HI		63	Poland	HI
19	Czech Republic	HI		64	Portugal	HI
20	Dominican Republic	MI		65	Qatar	HI
21	Ecuador	MI		66	Korea, Rep.	HI
22	Egypt, Arab Rep.	LI		67	Romania	MI
23	El Salvador	LI		68	Russian Federation	MI
24	Estonia	LI		69	Saudi Arabia	HI
25	Ethiopia	LI		70	Senegal	LI
26	Gabon	MI		71	Serbia	MI
27	Georgia	MI		72	Singapore	HI
28	Ghana	LI		73	Slovak Republic	HI
29	Greece	HI		74	Slovenia	HI
30	Grenada	MI		75	South Africa	MI
31	Guyana	MI		76	Sri Lanka	MI
32	Hungary	HI		77	Suriname	MI
33	Indonesia	LI		78	Tanzania	LI
34	Iran, Islamic Rep.	MI		79	Thailand	MI
35	Italy	HI		80	Togo	LI
36	Jamaica	MI		81	Tunisia	LI
37	Jordan	MI		82	Turkey	MI
38	Kazakhstan	MI		83	Turkmenistan	MI
39	Kenya	LI		84	Ukraine	LI
40	Kuwait	HI		85	U.A.E	HI
41	Kyrgyz Republic	LI		86	Uruguay	HI
42	Latvia	HI		87	Uzbekistan	LI
43	Lebanon	MI		88	Venezuela, RB	MI
44	Libya	MI		89	Vietnam	LI
45	Lithuania	HI		90	Zambia	LI

## Supporting information

S1 Data (ZIP)
